# Characterization of Antigen-Presenting Cell Subsets in Human Liver-Draining Lymph Nodes

**DOI:** 10.3389/fimmu.2019.00441

**Published:** 2019-03-14

**Authors:** Patrick P. C. Boor, Brenda M. Bosma, Khe T. C. Tran, Luc J. W. van der Laan, Hanneke Hagenaars, Jan N. M. IJzermans, Herold J. Metselaar, Jaap Kwekkeboom

**Affiliations:** ^1^Department of Gastroenterology and Hepatology, Erasmus MC–University Medical Centre, Rotterdam, Netherlands; ^2^Department of Surgery, Erasmus MC–University Medical Centre, Rotterdam, Netherlands

**Keywords:** myeloid dendritic cell, plasmacytoid dendritic cell, macrophage, human liver, immunological tolerance

## Abstract

T-cell immunity in the liver is tightly regulated to prevent chronic liver inflammation in response to antigens and toxins derived from food and intestinal bacterial flora. Since the main sites of T cell activation in response to foreign components entering solid tissues are the draining lymph nodes (LN), we aimed to study whether Antigen-Presenting Cell (APC) subsets in human liver lymph-draining LN show features that may contribute to the immunologically tolerant liver environment. Healthy liver LN, iliac LN, spleen and liver perfusates were obtained from multi-organ donors, while diseased liver LN were collected from explanted patient livers. Inguinal LN were obtained from kidney transplant recipients. Mononuclear cells were isolated from fresh tissues, and immunophenotypic and functional characteristics of APC subsets were studied using flowcytometry and in *ex vivo* cultures. Healthy liver-draining LN contained significantly lower relative numbers of CD1c^+^ conventional dendritic cells (cDC2), plasmacytoid DC (PDC), and CD14^+^CD163^+^DC-SIGN^+^ macrophages (MF) compared to inguinal LN. Compared to spleen, both types of LN contained low relative numbers of CD141^hi^ cDC1. Both cDC subsets in liver LN showed a more activated/mature immunophenotype than those in inguinal LN, iliacal LN, spleen and liver tissue. Despite their more mature status, cDC2 isolated from hepatic LN displayed similar cytokine production capacity (IL-10, IL-12, and IL-6) and allogeneic T cell stimulatory capacity as their counterparts from spleen. Liver LN from patients with inflammatory liver diseases showed a further reduction of cDC1, but had increased relative numbers of PDC and MF. In steady state conditions human liver LN contain relatively low numbers of cDC2, PDC, and macrophages, and relative numbers of cDC1 in liver LN decline during liver inflammation. The paucity of cDC in liver LN may contribute to immune tolerance in the liver environment.

## Introduction

The liver is continuously exposed to food components and bacterial products that enter the liver from the gastrointestinal tract via the portal vein. To prevent continuous liver inflammation in response to these gut-derived components, local immune responses in the liver are tightly regulated. Therefore, the liver environment favors induction of peripheral immune tolerance rather than immunity (1).

It has been hypothesized that dendritic cells (DC) are key players in maintaining the fine balance between T-cell responsiveness and unresponsiveness in the liver (2). Conventional DC (cDC) are the most highly specialized antigen-presenting cells (APC) and they play a critical role in the initiation and direction of T-cell responses (3, 4). Immature cDC are located throughout most body tissues and are specialized in the uptake and processing of antigens. Under steady state conditions there is a continuous migration of antigen-loaden cDC from non-lymphoid tissues through the lymphatics toward the draining lymph nodes (LN), where they present antigens that they acquired in their tissue of origin to T cells, and this process is accelerated in response to inflammatory signals. During migration to LN, cDC complete their maturation and acquire potent T cell stimulatory capacity by upregulation of MHC and co-stimulatory molecules (5). In addition to migratory cDC, LN contain resident cDC which emerge from circulating precursors and spend their entire lives within LN, where they acquire antigens from non-lymphoid tissue that in soluble form via lymphatic drainage enter LN, and present them to T cells (6).

Human cDC can be subdivided into two distinct ontogeneic lineages: type 1 cDC (cDC1) which express high levels of CD141 (BDCA3) and the necrotic cell receptor CLEC9a, and type 2 cDC (cDC2) that express CD1c (BDCA1) (7–9). Both cDC subsets are potent stimulators of CD4^+^ T-cell responses (10), but differ in stimulatory capacity of CD8^+^ T cells. cDC1 have a superior ability to cross-present antigens from necrotic cells to CD8^+^ T cells compared to cCD2 (11–14). The cross-presenting capacity of cDC2 is still unclear, although recent data suggest that they can cross-present soluble antigens (15).

Next to cDC, plasmacytoid DC (PDC) are a separate DC lineage. PDC are unique in rapidly producing massive amounts of type I interferon upon recognition of viral nucleotides or self-DNA-protein complexes by their Toll-Like receptors (TLR). Although PDC have weaker antigen presenting capacity than cDC, they can mature upon stimulation and prime CD4^+^ and CD8^+^ T cell responses (9, 16). On the other hand they can contribute to immunological tolerance by their capacity to generate CD4^+^ and CD8^+^ regulatory T cells (Treg) from naïve CD4^+^ or CD8^+^ T cells, respectively (17, 18).

Considerable efforts have been done to study whether DC in liver tissue possess unique characteristics that may contribute to immunological tolerance in the liver, but contradictory conclusions have been reached. On the one hand, mouse liver MDC were reported to have an immature phenotype and to be functionally compromised (19–22). On the other hand, however, other studies found no functional differences between distinct DC subsets in mouse liver and spleen, but reported differences in DC subset composition between both tissues (23, 24). In comparison to blood, human liver was shown to contain more cDC1 (25). In addition, human liver cDC2 produce higher amounts of the anti-inflammatory cytokine IL-10 compared to blood or skin MDC, although also being able to produce pro-inflammatory cytokines upon TLR-stimulation (26–28).

T cell activation by DCs is generally not initiated in parenchymal tissue, but in specific secondary lymphoid organs like the spleen or tissue-draining LN. In humans, major liver lymph vessels leave the liver at the hilus, and liver-derived lymph drains to LN situated at the hilus and along the hepatic artery and portal vein (29). Experimental animal studies have shown that cDC that migrate from the liver are trapped in liver-draining LN (30–32), and that T-cell activation to antigens delivered into the liver mainly occurs in these LN (32, 33). In humans, T cells specific for HBV antigens have been found to be enriched in liver-draining LN and not in the liver itself (34).

Despite the probable importance of hepatic LN in the regulation of T cell responses to antigens in the liver, very little is known about the composition and functions of APC in human liver lymph-draining LN. We therefore asked whether APC in liver LN differ from their counterparts in LN located at sites of the body where predominantly immunogenic responses are required, such as inguinal LN to which lymph from the legs is drained and which should be able to react to external pathogens upon disturbances of the skin barrier. The aim of this study was therefore to compare subset composition, phenotype and functions of the main known human APC subsets in human liver-draining LN with their counterparts in skin and muscle-draining inguinal LN. In addition, we compared APC subsets in liver LN with those in another secondary lymphoid tissue, i.e., spleen, and with those in human liver tissue, which contains cDC that migrate to liver LN.

## Tissues, Materials, and Methods

### Collection of Lymph Nodes, Spleen, and Liver Perfusate

Spleen tissues were obtained from multi-organ donors during organ donation procedures for the purpose of donor HLA-typing, and residual tissue was used for research. Iliac LN were collected for research purposes from 6 multi-organ donors during organ donation procedures. Hepatic LN were resected along the hepatic artery and portal vein in the porta hepatis from donor livers and diseased patient explant livers during liver transplantation procedures. Liver leukocytes were collected during routine perfusion of liver grafts immediately before transplantation, as we described previously (27). Inguinal LN were obtained from kidney transplant recipients during kidney transplantation procedures, and are considered as skin/muscle draining LN. The Ethics Committee of the Erasmus MC approved collection of the tissues for research purposes, and informed consent of each patient was obtained.

### Reagents

The following mAbs and reagents were used: IgG1-FITC, IgG1-PerCP-Cy5.5, IgG2a-APC, IgG1-PB, CD14-PE, CD14-PerCP, CD45-HorizonV500, streptavidin-PerCP, anti-IFNγ-FITC, 7AAD and Lineage cocktail-FITC (CD3, 16,19, 20,14, 56) from BD Biosciences, Erembodegem, Belgium; CD80-FITC from Beckman Coulter, Woerden, the Netherlands; anti-BDCA1-PE, anti-BDCA2-FITC, anti-BDCA2-biotin, anti-BDCA4-PE, anti-BDCA3-APC, anti-Clec9a-VioBlue, CD19 microbeads and anti-PE-microbeads from Miltenyi Biotec, Bergisch Gladbach, Germany; CD1a-PB, CD209 (DC-SIGN)-PECy7, CD206-eFluor450, CD40-APC and CD274 (PDL1)-PECy7 from eBioscience, Vienna, Austria; CD86-APC, CD86-PB, CD163-PerCP-Cy5.5, CD40-PerCP-Cy5.5, HLA DR-APC-Cy7 and CD209 (DC-SIGN)-APC from Biolegend, London, United Kingdom. PMA, ionomycin and brefeldin A are from Sigma-Aldrich, St Louis, MO. The fix&perm cell permeabilization kit was obtained from An der Grub, Vienna, Austria.

### Isolation of Mononuclear Cells (MNC)

Lymph nodes and spleen tissues were cut into small pieces and treated with 500 μg/ml collagenase type IV and 200 μg/ml DNAse I (both from Sigma Aldrich, Zwijndrecht, the Netherlands) at 37°C. After 20 min the collagenase reaction was stopped by adding fetal bovine serum and the tissue pieces were passed over a nylon mesh filter (100 μm pore diameter) to obtain single cell suspensions. To determine possible effects of collagenase on surface molecule expression, in preliminary experiments LN and spleen tissues were divided into two parts, and one part was treated with collagenase and the other part not. We observed that yields of MDC subsets were considerably increased upon isolations with collagenase, and therefore all data presented in the paper are derived from cells isolated by collagenase treatment, except data on liver cells because these were obtained using single cells collected from liver perfusates. MNC were obtained from single cell suspensions of tissue by Ficoll Paque (GE Healthcare Biosciences AB, Uppsala, Sweden) density gradient centrifugation. Cell yield and viability was determined by counting with trypan blue.

### Flowcytometry

Flowcytometric analysis was used to immunophenotype splenic, lymph node-, and liver APC. MNC were labeled with different antibody combinations. Appropriate isotype matched control antibodies were used to determine gates. APC subsets were determined within a MNC gate after exclusion of CD45^−^ non-hematopoietic cells and 7AAD^+^ dead cells. cDC subsets were distinguished on basis of CD11c, CD1c, and CD141 expression. PDC were defined as BDCA2^+^BDCA4^+^ cells, and monocytes/macrophages as CD14^+^. At least 500,000 events were acquired using a FACSCanto II flowcytometer and analyzed using FACSDiva 6.1 software (BD Biosciences) And Flowjo software (version 10.2, BD Biosciences).

### Purification of cDC2 and PDC

cDC2 were enriched from hepatic LN and spleen MNC by incubation with CD19, CD3, and CD15 microbeads and depletion of B and T cells and granulocytes by separation over a Large Depletion column using a MidiMACS separation device (Miltenyi Biotec, Bergisch Gladbach, Germany). Subsequently, the non-adherent cells were labeled with CD1c-PE, CD14-PerCP and CD20-Pacific Blue antibodies, and cDC2 were isolated by flowcytometric cell sorting by first excluding CD14^+^ and CD20^+^ cells, and subsequent selection of CD1c^+^ cells. Purity of the isolated cDC2 as determined by flowcytometry was typically >95%. To isolate PDC, LN MNC were labeled with anti-PE-conjugated anti-BDCA-4 antibody and anti-PE microbeads (Miltenyi Biotec) and purified by two round of separation over MS columns.

### Cytokine Production and T-Cell Stimulatory Capacity of DC

Purified cDC2 from hepatic LN and spleen from the same donors were cultured at a concentration of 2 × 10^4^ cells/200 ul in round bottom 96-well culture plates in RPMI1640 supplemented with 10% FCS (Hyclone, Logan, UT, USA), penicillin (100 U/ml), streptomycin (100 U/ml) and GM-CSF (500 U/ml; Leucomax, Novartis Pharma, Arnhem, the Netherlands) and stimulated with either 20 μg/ml synthetic double-stranded RNA (polyriboinosinic-polyribocytidylic acid; poly I:C; Sigma-Aldrich, St. Louis, MO) and 1,000 U/ml IFN-γ or CD40L-transfected J558 plasmacytoma cells (2 × 10^4^ cells/well) and 1,000 U/ml IFN-γ for 24 h and 37°C. After 24 h, supernatants were harvested and levels of IL-12, IL-6, and IL-10 were determined by standard enzyme-linked immunosorbent assay (ELISA) according to the manufacturer's instructions (eBioscience, Vienna, Austria).

The T cell stimulatory capacity of cDC2 or PDC was assessed by co-culturing purified MDC or PDC at different concentrations (10, 5, 2.5, 1.25 × 10^3^ cells/200 ul) in round bottom 96-well culture plates in RPMI1640 supplemented with 10% FCS, penicillin and streptomycin with 1.5 × 10^5^ CD3^+^ T cells isolated by magnetic selection using the pan T-cell isolation kit (Miltenyi Biotec) from blood of a healthy volunteer. In all experiments T cells from the same individual were used. After 5 days, T cell proliferation was assessed by measuring the incorporation of [^3^H]-thymidine (Radiochemical Center, Amersham, Little Chalfont, UK). 0.5 μCi was added per well and cultures were harvested 18 h later.

To determine their capacity of induce T-helper 1 responses, 1 × 10^5^ CD45RA^+^CD45RO^−^CD3^+^ naïve T cells obtained by flowcytometric cell sorting from blood of a healthy volunteer, were co-cultured with 1 × 10^4^ cDC2 isolated from hepatic LN or spleen. After 7 days T cells were re-stimulated with PMA (40 ng/ml) and ionomycin (1 ug/ml) for 6 h. During the last 5 h of restimulation brefeldin A (5 ug/ml) was added to inhibit protein transport processes. Intracellular IFN-γ expression was determined by using the Fix&perm cell permeabilization kit according to the manufacturer's instructions.

### Statistical Analysis

The Mann-Whitney *U*-test was used to test differences between tissues from different patients, and the Wilcoxon signed rank test was used for analysis of differences between paired tissues of the same donors. A *p* < 0.05 was considered significant. GraphPad Prism 5 software was used to perform the statistical tests.

## Results

### Characterization of Conventional Dendritic Cell Subsets in Lymphoid Organs and Liver

**Figure 1:**
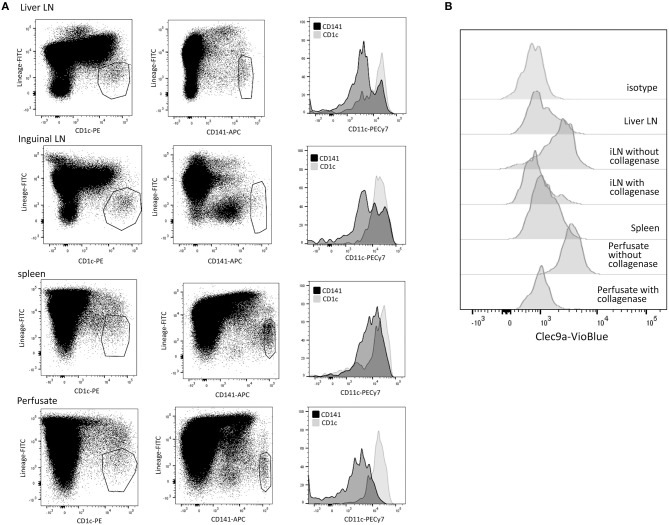
Characterization of cDC subsets in hepatic and inguinal lymph nodes, spleen, and liver. **(A)** Vital (7-AAD^−^)CD45^+^Lineage^−^CD1c^+^ and CD45^+^Lineage^−^CD141^+^cells were gated in MNC isolated from hepatic and inguinal lymph nodes, spleen and liver perfusate, and analyzed for CD11c expression. **(B)** Vital (7-AAD^−^)CD45^+^Lineage^−^CD141^bright^ cells were analyzed for Clec9A expression. Cells isolated from lymphoid tissues showed low Clec9A expression, while their counterparts in liver perfusate were Clec9A^+^. When liver perfusate cells were incubated with collagenase, Clec9A expression was lost. When leukocytes were isolated from inguinal LN without collagenase digestion, Clec9A was expressed on cDC2. iLN, inguinal LN.

To characterize DC subsets in the different tissues, MNC were isolated from freshly resected hepatic LN, inguinal LN, spleen, and liver graft perfusates, and analyzed for expression of CD45, CD11c, CD1c, CD141, and lineage markers (CD3, CD14, CD16, CD19, CD20, and CD56). Since leukocytes in liver graft perfusates accurately represent leukocytes present in liver tissue (27, 28, 35–37) we will refer to liver perfusate DC as liver DC. First, we analyzed CD11c expression on CD45^+^Lineage^−^CD1c^+^ and CD45^+^Lineage^−^CD141^+^ cells. We observed that lineage^−^CD1c^+^ cells express high levels of CD11c, while part of lin^−^CD141^hi^ cells were CD11c^dim^ in the different tissues (Figure 1A). In accordance with previous publications on cDC subsets in human tissues (9, 14), we concluded that CD141^hi^ cDC1 have variable CD11c expression. Therefore defined cDC1 as lin^−^CD141^hi^ cells and cDC2 as lin^−^CD11c^+^CD1c^+^ cells. Lin^−^CD141^hi^ cells from liver perfusate also expressed Clec9A, identifying them as bona fide cDC1 (13, 14). However, Clec9A expression was reduced or absent on cDC1 in lymphoid tissues (Figure 1B). This appeared to be due to the collagenase digestion used to isolate single cells from lymphoid tissues, which was not required for liver perfusate. Incubation of liver-derived leukocytes with collagenase resulted in loss of Clec9A expression on liver-derived cDC1, while isolation of single cells from LN without collagenase digestion resulted in cDC1 with clear Clec9A expression (Figure 1B). In none of the tissues cDC1 expressed CD1a, CD206, or DC-SIGN (data not shown), indicating that both types of LN, as well as spleen and liver, contain a homogeneous population of cDC1. In contrast, in all examined tissues 10–20% of cDC2 expressed CD1a and a small proportion expressed CD206 (data not shown), suggesting that a minority of cDC1 may represent migratory DC (38).

### Liver LN Contain Relatively Low Numbers of cDC2

**Figure 2:**
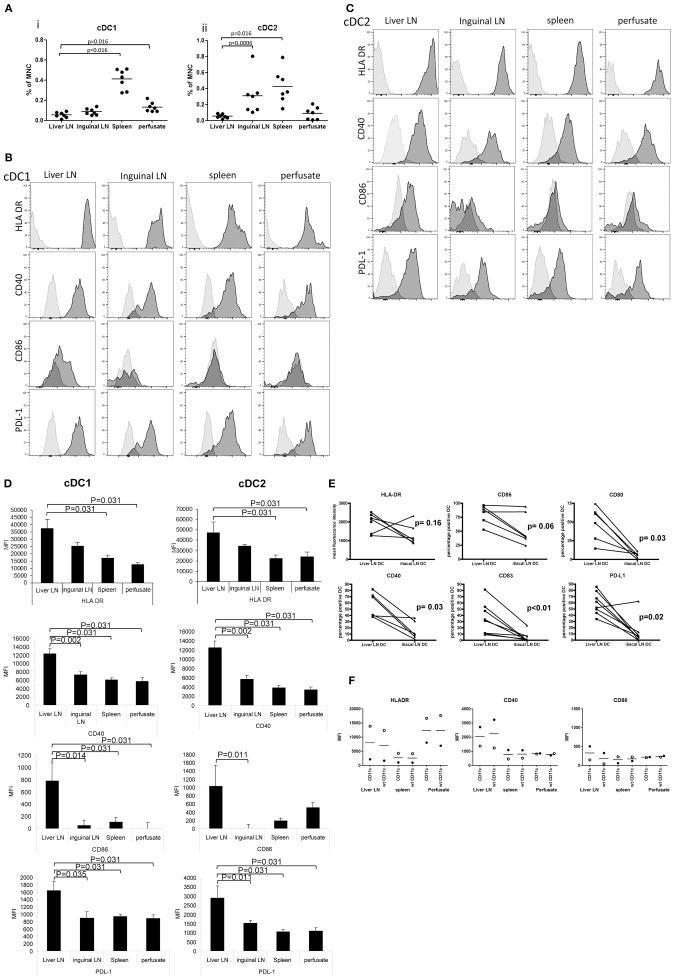
Relative cDC subset numbers and their maturation status in hepatic and inguinal lymph nodes, spleen and liver. **(A)** The proportions of lineage^−^CD141^bright^cDC1 and lineage^−^CD11c^+^CD1c^+^ cDC1were determined within CD45^+^ MNC. Dead cells were excluded from the analysis by excluding 7AAD^+^ cells. Dots represent individual tissues, and lines indicate mean values. Hepatic LN, spleen tissues, and liver perfusates were obtained from the same multi-organ donors, while inguinal LN were collected from kidney transplant recipients. **(B)** Histograms showing expression of HLA DR, CD40, CD86, and PDL-1 on lineage^−^CD141^bright^cDC1 from hepatic lymph nodes, inguinal lymph nodes, spleen, and liver. Light gray histograms are isotype control stains. **(C)** Histograms showing expression of HLA DR, CD40, CD86, and PDL-1 on lineage^−^CD1c^+^cDC2 in hepatic lymph nodes, inguinal lymph nodes, spleen, and liver. Light gray histograms are isotype control stains. **(D)** Summary of expression levels (MFI) of HLA DR, CD40, CD86, and PDL-1 on lineage^−^CD141^bright^cDC1 and lineage^−^CD1c^+^cDC2 in liver LN (*n* = 6) and inguinal lymph nodes (*n* = 7), spleen (*n* = 6) and liver (*n* = 6). Data are depicted as means ± SEM. **(E)** Expression levels of HLA-DR, CD86, CD80, CD40, CD83, and PDL-1 was determined on cDC2 in MNC isolated from paired liver LN and iliac LN from the same multi-organ donors (*n* = 6). **(F)** Comparison of the expression levels of HLA-DR, CD40, and CD86 on cDC2 in tissues of 2 different liver transplant donors gated as lineage^−^CD11c^+^CD1c^+^ (CD11c) cells or as lineage^−^CD1c^+^ without inclusion of CD11c in the gating strategy (wt).

To compare relative numbers of cDC subsets in the different tissues, we quantified proportions of lineage^−^CD141^bright^ cDC1 and lineage^−^CD11c^+^CD1c^+^ cDC2 within CD45^+^ cells. Of all tissues, spleen contained the highest proportions of both cDC subsets (Figure 2A). Interestingly, hepatic LN contained 6 times lower proportions of cDC1 compared to inguinal LN (0.053% vs. 0.31%; *p* = 0.0006), while both types of LN contained small but similar proportions of cDC2. These data demonstrate a selective reduction of the relative numbers of the cDC2 subset in human liver LN.

### Both cDC Subsets in Liver LN Are Highly Activated

To compare the maturation status of both cDC subsets in the different tissues, we analyzed their expression of HLA-DR, the co-stimulatory molecules CD40 and CD86, and the co-inhibitory molecule PDL-1. Because we had no more than 8 fluorochrome channels available on our FACS, we omitted CD11c from our gating strategy, and defined cDC2 cells for this analysis as lineage^−^CD1c^+^. Nevertheless, all cDC2 identified by this gating strategy were HLA-DR^+^, like cDC1 (Figures 2B,C). Expression levels of HLA-DR on both cDC subsets was higher in liver LN compared to spleen and liver (Figures 2B–D). Even more interestingly, in hepatic LN both cDC subsets expressed significantly higher levels of CD40, CD86 and PDL-1 compared to their counterparts in inguinal LN, spleen and liver, indicating that both cDC subsets in hepatic LN are in a more activated/mature status. To exclude that the observed differences in maturation status were confounded by possible differences between multi-organ donors (from which hepatic LN, spleens and liver perfusates were collected) and kidney transplant recipients (from which inguinal LN were obtained), paired comparisons of expression of HLA-DR, CD40, CD80, CD83, CD86, and PDL-1 on cDC2 in hepatic and iliac LN derived from the same multi-organ donors were performed. The data derived from these measurements confirm that cDC2 in hepatic LN have a more mature/activated immunophenotype compared to their counterparts in non-liver-draining LN (Figure 2E). Finally, we analyzed HLA-DR, CD40, and CD86 expression on cDC2 in 2 additional sets of tissues using antibody panels in which we replaced PD-L1 antibody by CD11c antibody, allowing for gating of cDC2 as lineage^−^CD11c^+^CD1c^+^ cells. This gating strategy yielded similar results for expression levels of HLA-DR and co-stimulatory molecules as the gating strategy without CD11c (Figure 2F).

### cDC2 in Hepatic Lymph Nodes Are Functionally Similar to Those in Spleen

**Figure 3:**
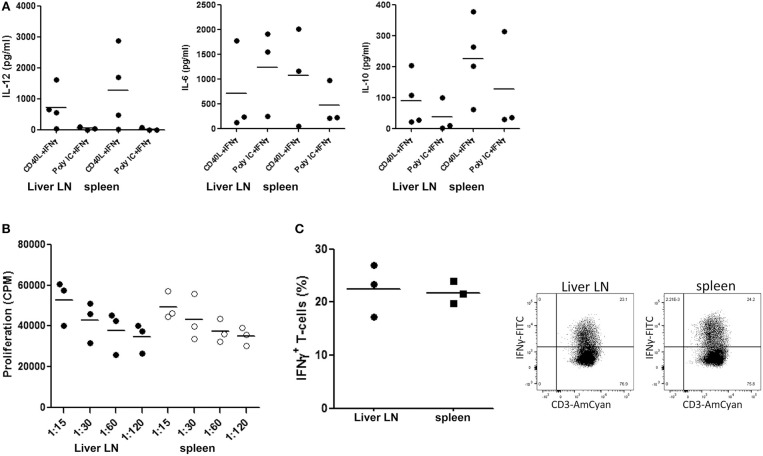
cDC2 isolated from hepatic lymph nodes and spleen are functionally similar. **(A)** cDC2 were purified from hepatic LN and spleen MNC from the same donors by immunomagnetic depletion of granulocytes, T cells and B cells followed by flowcytometric sorting of CD1c^+^CD14^−^CD20^−^ cells as described in the Methods section, and 2 × 10^4^ cells/200 μl were stimulated with either CD40L-transfected plasmacytoma cells (2 × 10^4^ J558 cells) and 1,000 U/ml IFN-γ, or with 20 μg/ml poly IC and 1,000 U/ml IFN-γ for 24 h at 37°C. After 24 h, supernatants were harvested and levels of IL-12, IL-6 and IL-10 were determined. Dots represent data from individual tissues, and lines indicate mean values. **(B)** Graded numbers of hepatic LN (solid symbols) and spleen (open symbols) cDC2 from the same donors were co-cultured with graded numbers of allogeneic T cells (from one batch) and T-cell proliferation was assessed after 5 days by [^3^H]-thymidine incorporation. Dots represent data from individual tissues and lines indicate mean values. **(C)** Naïve allogeneic CD3^+^CD45RA^+^ T cells were co-cultured with cDC2 purified from hepatic LN or spleen from the same donors. After 7 days the T-cells were re-stimulated with PMA and ionomycin for 6 h. For the last 5 h brefeldin A was added. Representative dot plots of intracellular expression of IFN-γ in CD3^+^ T cells are shown, and a summary of the results from experiments with cDC2 purified from tissues of 3 different donors is depicted. Dots represent data from individual tissues and lines indicate mean values.

We compared the functional properties of the most prominent cDC-subset present in hepatic LN, i.e., the cDC2, with those of its counterpart in spleen. cDC2 were purified from hepatic LN and spleen MNC obtained from the same donors and we analyzed their cytokine production upon *ex vivo* stimulation with CD40L plus IFN-γ or TLR3-agonist poly I:C plus IFN-γ. Since synergistic stimulation via TLR or CD40 with IFN-γ is required to induce maximal IL-12 production by human cDC2 (39), we added IFN-γ in all conditions. The cells secreted the pro-inflammatory cytokines IL-12 and IL-6 as well as the immune-regulatory cytokine IL-10 without apparent differences between those derived from hepatic LN and spleen (Figure 3A).

In addition, we compared their T-cell stimulatory capacity. Despite their more mature immunophenotype, cDC2 from liver LN demonstrated a comparable capacity to stimulate proliferation of allogeneic T-cells as their counterparts isolated from spleen (Figure 3B). Moreover, cDC2 from hepatic LN and spleen induced IFN-γ production in naïve allogeneic T-cells (Figure 3C) to a comparable extent.

### Liver LN Contain Relatively Low Numbers of Plasmacytoid Dendritic Cells

**Figure 4:**
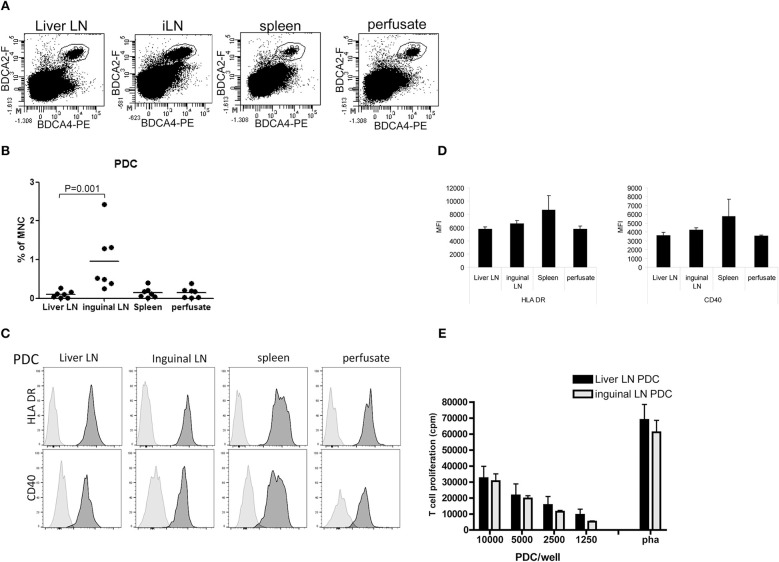
PDC in hepatic and inguinal lymph nodes, spleen and liver. **(A)** Flowcytometric determination of PDC in MNC isolated from hepatic and inguinal lymph nodes, spleen, and liver perfusate using anti-BDCA2 and anti-BDCA4 antibodies. Non-hematopoietic cells and dead cells were excluded from the analysis by exclusion of CD45^−^ cells and 7-AAD^+^ cells. **(B)** Percentages of BDCA2^+^BDCA4^+^ PDC within MNC in the different tissues. Dots represent data from individual tissues and lines indicate mean values. **(C)** Histograms showing HLA-DR and CD40 expression on PDC from the different tissues. Light gray histograms are isotype control stains. **(D)** Summary of the expression levels of HLA-DR and CD40 on PDC in hepatic (H) lymph nodes (*n* = 6) and inguinal lymph nodes (*n* = 7), spleen (*n* = 6), and liver (*n* = 6). Data are depicted as means with SEM. No significant differences were observed **(E)** Graded numbers of purified hepatic (*n* = 6) and inguinal LN PDC (*n* = 5) were co-cultured with allogeneic T-cells (from one batch) and T-cell proliferation was assessed after 5 days by [^3^H]-thymidine incorporation. T-cell proliferation in response to PHA is depicted as positive control condition. Data represent means with SEM. No significant differences were observed.

Relative PDC numbers within CD45^+^ MNC of the different tissues were determined using anti-BDCA2 and anti-BDCA4 antibodies (Figure 4A). Hepatic LN contained on average 0.13% PDC, which is comparable to the relative numbers of PDC in spleen (0.14%) and liver (0.19%), but ~7 times lower than PDC numbers in inguinal LN (0.95%; Figure 4B). The maturation status of PDC was assessed by determination of the expression of HLA-DR, CD40, CD86, and PDL-1. In none of the examined tissues CD86 expression was detected on PDC, and PDL-1 expression was very low (data not shown). CD40 and HLA-DR expression on PDC did not differ between the tissues (Figures 4C,D). In addition, PDC isolated from liver LN and inguinal LN showed comparable *ex vivo* allogeneic T-cell stimulatory capacity (Figure 4E).

### Liver LN Contain Relatively Low Numbers of CD14^+^DC-SIGN^+^Macrophages

**Figure 5:**
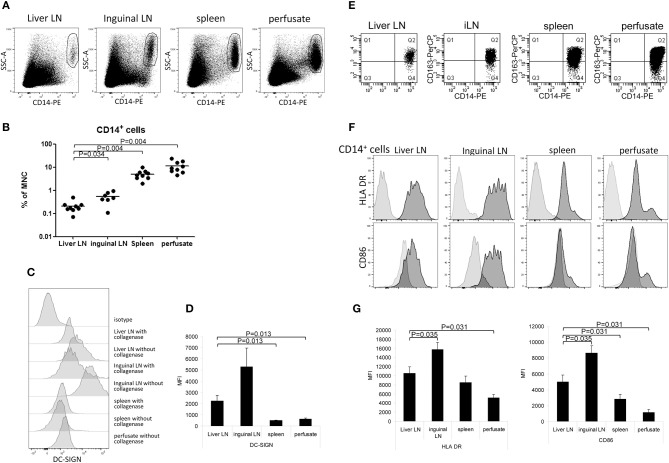
Relative CD14^+^ cell numbers and their activation status in hepatic and inguinal lymph nodes, spleen, and liver. **(A)** CD14^+^ cells were determined within vital CD45^−^ MNC. Representative dot plots are shown. **(B)** A summary of the percentages of CD14^+^ cells within vital CD45^−^ MNC isolated from the different tissues. Data from individual tissues are depicted as dots and lines indicate mean values on a log-scale. **(C)** DC-SIGN-expression on CD14^+^ cells that were isolated from tissues with collagenase or without collagenase treatment. Tissues were divided into two parts, and after cutting into small pieces, one part was treated with collagenase and the other part not. After that, single cell suspensions were made from both parts. **(D)** Summary showing DC-SIGN expression levels on CD14^+^ cells from liver lymph nodes (*n* = 6) and inguinal lymph nodes (*n* = 7), spleen (*n* = 6), and liver (*n* = 6). Except for liver, MNC were isolated from the tissues using collagenase. Data are depicted as means with SEM. **(E)** CD163 expression on CD14^+^ cells in MNC from liver LN, inguinal LN, spleen and liver perfusate. **(F)** Histograms showing HLA-DR and CD86 expression on CD14^+^ cells in the different tissues. Light gray histograms are isotype control stains. **(G)** Summary of HLA-DR and CD86 expression levels on CD14^+^ cells in liver lymph nodes (*n* = 6), inguinal lymph nodes (*n* = 7), spleen (*n* = 6), and liver (*n* = 6). Data are depicted as means with SEM.

In addition to the two MDC-subsets and PDC, we detected CD14^+^ cells in spleen and liver (5.0 ± 0.5% and 11.3 ± 2.8% of total CD45^+^MNC, respectively). In contrast, these cells were rare in both types of LN, but hepatic LN contained even 2-fold lower relative numbers compared to inguinal LN (0.23 ± 0.06% and 0.53 ± 0.03% of CD45^+^MNC, respectively; Figures 5A,B). CD14^+^ cells in both types of LN expressed much higher levels of DC-SIGN compared to those in spleen and liver (Figures 5C,D). In preliminary experiments in which we compared MNC isolations from LN or spleen with or without collagenase treatment, we observed that DC-SIGN expression was affected by collagenase digestion of the tissue (Figure 5B). Therefore, the expression levels of DC-SIGN on CD14^+^ cells isolated after collagenase digestion depicted in Figure 5D are probably under-estimations, except for liver cells which were always obtained as single cells present in liver perfusates and therefore not treated with collagenase. In all examined tissues the majority of CD14^+^ cells expressed CD163 (Figure 5E), but not CD206 (data not shown), suggesting that these cells represent macrophages (38, 40). Liver CD14^+^ cells represent most probably Kupffer cells, which have been shown to express low levels of DC-SIGN (41). The majority of CD14^+^DC-SIGN^+^CD206^−^ cells in LN have been shown to represent subcapsular macrophages (42, 43), while about one third expressed CD169 in addition to DC-SIGN (data not shown) and may therefore represent medullary sinus macrophages (43, 44). No CD14^+^CD169^+^ cells were found in spleen and liver. The activation status of the CD14^+^ cells was determined by the expression of HLA DR, CD80 and CD86. None of CD14^+^ cells in the examined tissues expressed CD80. Compared to CD14^+^ cells in inguinal LN, CD14^+^ cells in hepatic LN showed lower expression levels of HLA-DR and CD86, indicating a less activated status of these cells, although the presence of different macrophage sub-populations in the two types of LN cannot be excluded (Figures 5F,G).

### Decreased Numbers of cDC Subsets but Increased Numbers of Macrophages and PDC in Lymph Nodes of Diseased Livers

**Figure 6:**
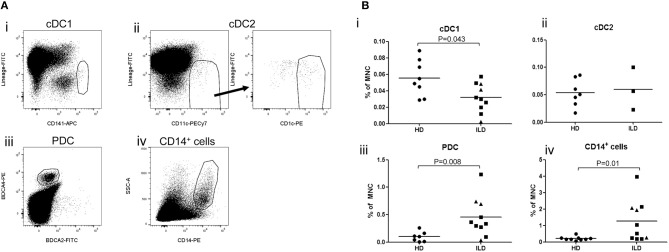
**(A)** Relative numbers of APC subsets in liver lymph nodes of patients with inflammatory liver diseases. Flowcytometric analysis of cDC1 and cDC2 in single cell suspension of a LN attached to an explanted liver of a PSC patients. Within viable CD45^+^ cells, CD141^hi^ cDC1 and CD11c^+^CD1c^+^ cDC2 were gated. **(B)**The percentages of cDC1, cDC2, PDC, and CD14^+^ cells were determined within CD45^+^ MNC in hepatic lymph nodes derived from healthy donors (HD; *n* = 8) and patients with inflammatory liver disease (ILD; *n* = 10 for cDC1 and *n* = 3 for cDC2). Patients with primary sclerosing cholangitis are depicted as squares and patients with auto-immune hepatitis as triangles. Lines indicate means.

Finally, we studied whether inflammatory liver disease affects numbers of the different APC-subsets in hepatic LN. For this purpose, we isolated MNC from freshly resected liver LN collected during liver transplant procedures from explant livers of patients with primary sclerosing cholangitis or auto-immune hepatitis, and compared relative numbers of the different APC subsets within CD45^+^ MNC with those in liver LN isolated from healthy livers of multi-organ donors. Interestingly, relative numbers of cDC1 were decreased, while relative numbers of macrophages and PDC were increased in hepatic LN of patients with inflammatory liver disease compared to hepatic LN from healthy liver (Figures 6A,B).

## Discussion

Previous studies have shown that human LN contain two main cDC subsets which correspond to the two main cDC subsets in blood: CD141^bright^ cDC1 and CD1c^+^cDC2 and (14, 38). The present study shows that the same cDC subsets are present in liver LN. However, cDC2 are present at much lower relative numbers in hepatic LN compared to skin/muscle-draining inguinal LN and spleen, while cDC1 are a minor subset in both hepatic and inguinal LN. Like cDC1, cDC2 can stimulate both CD4^+^ and CD8^+^ T cells (10), but cDC2 can produce IL-12 while cDC1 cannot (39, 45). Since IL-12 is a main driver of T-helper 1 differentiation and cytotoxic CD8^+^ T cell responses, the reduced numbers of cDC2 in hepatic LN may contribute to the relatively low immunogenicity of the liver environment.

Interestingly, both cDC subsets displayed a more mature phenotype with higher expression of co-stimulatory molecules in hepatic LN compared to inguinal LN, iliacal LN, spleen, and liver. This observation is in line with a previous study showing that rat liver lymph contains highly mature MDC (46). The enhanced maturation status of cDC in hepatic LN may be caused by stimulation by bacterial products, such as endotoxins, that continuously enter the liver from the intestinal tract via the portal circulation. Such stimulation may have occurred in the liver before migration of cDC to the sentinel LN or in the LN. Nevertheless, cDC2 isolated from hepatic LN showed a comparable T-cell stimulatory capacity, cytokine production and capacity to induce IFNγ production in naïve T-cells compared to those isolated from spleen. We speculate that the enhanced expression of the co-inhibitory ligand PDL-1 on MDC in hepatic LN may compensate for the enhanced expression of co-stimulatory molecules in T-cell stimulation. The equal capacity of hepatic LN and splenic cDC2 to induce IFNγ-production in naïve T cells is probably associated with their similar capacity to produce IL-12. Our current data show that cDC2 in human liver LN are not functionally compromised, as has been suggested previously for cDC in mouse liver (19, 20, 22). Accordingly, we have previously shown that cDC2 isolated from human liver tissue are able to produce pro-inflammatory cytokines and acquire T-cell stimulatory capacity upon TLR-activation (28).

We expected that liver inflammation would enhance migration of cDC to the draining LN, and thereby cause increased numbers of cDC in hepatic LN. Indeed, induction of inflammation in the skin by local injection of a TLR-9 agonist and GM-CSF into the scar of resected melanoma resulted in selectively increased numbers of cDC1 in sentinel lymph nodes (47). Moreover, inflammatory liver disease is associated with depletion of cDC1 from human liver, and is was hypothesized that they might have migrated to hepatic LN due to inflammatory stimuli (25). In contrast to our expectation, relative numbers of cDC1 were reduced in LN attached to inflamed livers compared to those in LN of healthy livers. We hypothesize that the decreased relative numbers of cDC1 in hepatic LN in these patients might be caused by activation-induced cell death or, alternatively, that liver cirrhosis impedes migration of MDC through lymphatics to the regional LN. It is tempting to speculate that the paucity of cDC1 in sentinel liver LN in end-stage liver diseases may lead to a further mitigation of T-cell responses to liver-derived antigens compared to healthy livers.

We confirm our previous finding that hepatic LN (48) contain much lower numbers of PDC than skin/muscle draining inguinal LN. However, we now show that PDC from both types of LN do not differ in terms of activation/maturation status and T-cell stimulatory capacity. Differences in migration route between MDC and PDC may explain the similar maturation status of PDC in hepatic and inguinal LN. PDC do not migrate via the lymph to LN, but migrate from the blood circulation into LN via the high endothelial venules, and are mainly attracted by the CCR-7 ligands CCL19 and CCL21 (49, 50). Therefore, they are probably not exposed to maturation stimuli present in the liver environment, like cDC. PDC are important in anti-viral defense by production of IFN-α, amongst others against HCV (51, 52), but can also present antigens to T-cells. They are able to prime productive effector T-cell responses (9, 16), but can also induce differentiation of CD4^+^ as well as CD8^+^ Treg (17, 18). The circumstances in which the balance tips toward activation of effector T cells or Treg are not well-understood, and probably depend on the micro-environmental conditions in the tissue in which they reside. In addition to their capacity to directly activate T cells, PDC interact with cDC to enhance their T-cell stimulatory capacity (53, 54). Together, it is difficult to speculate on the immunological consequences of the low numbers of PDC in hepatic LN under steady state conditions. However, in contrast to cDC, numbers of PDC are increased in sentinel LN of inflamed livers with cholestatic or auto-immune diseases. Apparently, hepatic LN can “normalize” their PDC content during liver inflammation. We speculate that liver inflammation leads to increased production of CCL19 and CCL21 and/or other chemo-attractants in hepatic LN, that induce migration of PDC into LN.

In addition to cDC and PDC, also macrophages are able to present antigens to T cells although less efficiently (55). Liver LN were found to contain 2-fold lower numbers of CD14^+^ macrophages than in skin/muscle draining inguinal LN. Based on their immunophenotype, the majority of these cells are probably subcapsular macrophages, whose main function is to capture lymph-born viral particles and present them to B-cells (56). However, they are also able to cross-present lymph-derived antigens to CD8^+^ T cells (57, 58), and promote activation of NK-cells, NKT-cells, PDC and γδ-T-cells, possibly via production of type I IFN, IL12 and IL18 (59). In contrast to cDC, CD14^+^ cells in hepatic LN showed a reduced maturation status compared to their counterparts in inguinal LN. The origin of the macrophages in liver LN is currently unknown. One possibility is that (a subset of) Kupffer cells migrate via lymph to hepatic LN, but in rat it has been shown that liver lymph does not contain Kupffer cells (30). Since macrophages are also present in inguinal LN, even in higher numbers, we favor the hypothesis that these macrophages derived from circulating monocytes. This hypothesis would also explain the relatively low activation status of these cells in liver LN, because they have, like PDC, not been exposed to endotoxins present in the liver. It can also explain the increase in CD14^+^ cells in lymph nodes during inflammatory conditions, since monocyte chemoattractant protein (MCP)-1 increases at the luminal surface of high endothelial venules during inflammation, which enhances the migration of CD14^+^ cells into LN (60).

The strength of this study is that we are the first to comprehensively characterize APC subsets in human liver lymph nodes. Limitations of our study are that: (1) We could not study the functional properties of cDC1 in liver LN, because it was impossible to isolate sufficient numbers of this rare subset; (2) Since the numbers of fluorochrome channels on our flowcytometer were limited to 8, we could not include CD11c in all phenotypic analysis of cDC2. However, main findings on the phenotypes of cDC2 were confirmed in measurements that included CD11c in the gating strategy.

In summary, we observed that in steady state conditions human liver LN contain relatively low numbers of cDC2, PDC, and subcapsular macrophages. Whereas, numbers of cDC1 further decrease during liver inflammation and cirrhosis, numbers of PDC and subcapsular macrophages increase, suggesting important contributions of these APC cell subsets to liver immunity. Functional studies are required to reveal whether these APC cell subsets do play a role in regulating liver immunity. Importantly, we found that cDC2 in hepatic LN are not dysfunctional. The paucity of both main subsets of cDC subsets in liver LN, both during steady state conditions and during liver inflammation, may contribute to the immunologically tolerant liver environment.

## Author Contributions

PB contributed to study concept and design, acquisition, analysis and interpretation of data, statistical analysis, drafting of the manuscript, critical revision of the manuscript for important intellectual content. BB contributed to study concept and design, acquisition, analysis, and interpretation of data. KT and JI contributed to material support and critical revision of the manuscript for important intellectual content. LvdL contributed to critical revision of the manuscript for important intellectual content. HM obtained funding and contributed to critical revision of the manuscript for important intellectual content. HH contributed to material support. JK contributed to study concept and design, interpretation of data, drafting of the manuscript, critical revision of the manuscript for important intellectual content, and study supervision.

### Conflict of Interest Statement

The authors declare that the research was conducted in the absence of any commercial or financial relationships that could be construed as a potential conflict of interest.

## References

[1] Crispe IN Giannandrea M Klein I John B Sampson B Wuensch S. Cellular and molecular mechanisms of liver tolerance. Immunol Rev. (2006) 213:101–18. 10.1111/j.1600-065X.2006.00435.x 16972899

[2] Lau AH Thomson AW. Dendritic cells and immune regulation in the liver. Gut. (2003) 52:307–14. 10.1136/gut.52.2.307 12524419 PMC1774973

[3] Hart DN. Dendritic cells: unique leukocyte populations which control the primary immune response. Blood. (1997) 90:3245–87. 9345009

[4] Banchereau J Steinman RM. Dendritic cells and the control of immunity. Nature. (1998) 392:245–52. 10.1038/32588 9521319

[5] Worbs T Hammerschmidt SI Forster R. Dendritic cell migration in health and disease. Nat Rev Immunol. (2017) 17:30–48. 10.1038/nri.2016.116 27890914

[6] Eisenbarth SC. Dendritic cell subsets in T cell programming: location dictates function Nat Rev Immunol. (2019) 19:89–103. 10.1038/s41577-018-0088-1 30464294 PMC7755085

[7] Guilliams M Dutertre CA Scott CL McGovern N Sichien D Chakarov S. Unsupervised high-dimensional analysis aligns dendritic cells across tissues and species. Immunity. (2016) 45:669–84. 10.1016/j.immuni.2016.08.015 27637149 PMC5040826

[8] Heidkamp GF Sander J Lehmann CHK Heger L Eissing N Baranska A Human lymphoid organ dendritic cell identity is predominantly dictated by ontogeny, not tissue microenvironment. Sci Immunol. (2016) 1: eaai7677 10.1126/sciimmunol.aai7677 28783692

[9] Alcantara-Hernandez M Leylek R Wagar LE Engleman EG Keler T Marinkovich MP. High-dimensional phenotypic mapping of human dendritic cells reveals interindividual variation and tissue specialization. Immunity. (2017) 47:1037–50 e1036. 10.1016/j.immuni.2017.11.001 29221729 PMC5738280

[10] O'Keeffe M Mok WH Radford KJ. Human dendritic cell subsets and function in health and disease. Cell Mol Life Sci. (2015) 72:4309–25. 10.1007/s00018-015-2005-0 26243730 PMC11113503

[11] Huysamen C Willment JA Dennehy KM Brown GD. CLEC9A is a novel activation C-type lectin-like receptor expressed on BDCA3+ dendritic cells and a subset of monocytes. J Biol Chem. (2008) 283:16693–701. 10.1074/jbc.M709923200 18408006 PMC2562446

[12] Bachem A Guttler S Hartung E Ebstein F Schaefer M Tannert A. Superior antigen cross-presentation and XCR1 expression define human CD11c+CD141+ cells as homologues of mouse CD8+ dendritic cells. J Exp Med. (2010) 207:1273–81. 10.1084/jem.20100348 20479115 PMC2882837

[13] Jongbloed SL Kassianos AJ McDonald KJ Clark GJ Ju X Angel CE. Human CD141+ (BDCA-3)+ dendritic cells (DCs) represent a unique myeloid DC subset that cross-presents necrotic cell antigens. J Exp Med. (2010) 207:1247–60. 10.1084/jem.20092140 20479116 PMC2882828

[14] Haniffa M Shin A Bigley V McGovern N Teo P See P. Human tissues contain CD141hi cross-presenting dendritic cells with functional homology to mouse CD103+ nonlymphoid dendritic cells. Immunity. (2012) 37:60–73. 10.1016/j.immuni.2012.04.012 22795876 PMC3476529

[15] Chiang MC Tullett KM Lee YS Idris A Ding Y McDonald KJ. Differential uptake and cross-presentation of soluble and necrotic cell antigen by human DC subsets. Eur J Immunol. (2016) 46:329–39. 10.1002/eji.201546023 26542182

[16] Villadangos JA Young L. Antigen-presentation properties of plasmacytoid dendritic cells. Immunity. (2008) 29:352–61. 10.1016/j.immuni.2008.09.002 18799143

[17] Matta BM Castellaneta A Thomson AW. Tolerogenic plasmacytoid DC. Eur J Immunol. (2010) 40:2667–76. 10.1002/eji.201040839 20821731 PMC3974856

[18] Boor PP Metselaar HJ Jonge S Mancham S van der Laan LJ Kwekkeboom J. Human plasmacytoid dendritic cells induce CD8(+) LAG-3(+) Foxp3(+) CTLA-4(+) regulatory T cells that suppress allo-reactive memory T cells. Eur J Immunol. (2011) 41:1663–74. 10.1002/eji.201041229 21469126

[19] Rastellini C Lu L Ricordi C Starzl TE Rao AS Thomson AW. Granulocyte/macrophage colony-stimulating factor-stimulated hepatic dendritic cell progenitors prolong pancreatic islet allograft survival. Transplantation. (1995) 60:1366–70. 8525540 PMC2966312

[20] Khanna A Morelli AE Zhong C Takayama T Lu L Thomson AW. Effects of liver-derived dendritic cell progenitors on Th1- and Th2-like cytokine responses *in vitro* and *in vivo*. J Immunol. (2000) 164:1346–54. 10.4049/jimmunol.164.3.1346 10640749

[21] Morelli AE O'Connell PJ Khanna A Logar AJ Lu L Thomson AW. Preferential induction of Th1 responses by functionally mature hepatic (CD8alpha- and CD8alpha+) dendritic cells: association with conversion from liver transplant tolerance to acute rejection. Transplantation. (2000) 69:2647–57. 10.1097/00007890-200006270-00027 10910289

[22] O'Connell PJ Morelli AE Logar AJ Thomson AW. Phenotypic and functional characterization of mouse hepatic CD8 alpha+ lymphoid-related dendritic cells. J Immunol. (2000) 165:795–803. 10.4049/jimmunol.165.2.795 10878353

[23] Lian ZX Okada T He XS Kita H Liu YJ Ansari AA. Heterogeneity of dendritic cells in the mouse liver: identification and characterization of four distinct populations. J Immunol. (2003) 170:2323–30. 10.4049/jimmunol.170.5.2323 12594254

[24] Pillarisetty VG Shah AB Miller G Bleier JI DeMatteo RP. Liver dendritic cells are less immunogenic than spleen dendritic cells because of differences in subtype composition. J Immunol. (2004) 172:1009–17. 10.4049/jimmunol.172.2.1009 14707074

[25] Kelly A Fahey R Fletcher JM Keogh C Carroll AG Siddachari R. CD141(+) myeloid dendritic cells are enriched in healthy human liver. J Hepatol. (2014) 60:135–42. 10.1016/j.jhep.2013.08.007 23968887

[26] Goddard S Youster J Morgan E Adams DH. Interleukin-10 secretion differentiates dendritic cells from human liver and skin. Am J Pathol. (2004) 164:511–9. 10.1016/S0002-9440(10)63141-0 14742257 PMC1602266

[27] Bosma BM Metselaar HJ Mancham S Boor PP Kusters JG Kazemier G. Characterization of human liver dendritic cells in liver grafts and perfusates. Liver Transpl. (2006) 12:384–93. 10.1002/lt.20659 16498646

[28] Bosma BM Metselaar HJ Gerrits JH van Besouw NM Mancham S Groothuismink ZM. Migration of allosensitizing donor myeloid dendritic cells into recipients after liver transplantation. Liver Transpl. (2010) 16:12–22. 10.1002/lt.21961 19866483

[29] Graham NJ Libshitz HI. Cascade of metastatic colorectal carcinoma from the liver to the anterior diaphragmatic lymph nodes. Acad Radiol. (1995) 2:282–5. 10.1016/S1076-6332(05)80185-0 9419563

[30] Matsuno K Ezaki T Kudo S Uehara Y. A life stage of particle-laden rat dendritic cells in vivo: their terminal division, active phagocytosis, and translocation from the liver to the draining lymph. J Exp Med. (1996) 183:1865–78. 10.1084/jem.183.4.1865 8666943 PMC2192479

[31] Kudo S Matsuno K Ezaki T Ogawa M. A novel migration pathway for rat dendritic cells from the blood: hepatic sinusoids-lymph translocation. J Exp Med. (1997) 185:777–84. 10.1084/jem.185.4.777 9034155 PMC2311511

[32] Barbier L Tay SS McGuffog C Triccas JA McCaughan GW Bowen DG. Two lymph nodes draining the mouse liver are the preferential site of DC migration and T cell activation. J Hepatol. (2012) 57:352–8. 10.1016/j.jhep.2012.03.023 22542491

[33] Wrenshall LE Ansite JD Eckman PM Heilman MJ Stevens RB Sutherland DE. Modulation of immune responses after portal venous injection of antigen. Transplantation. (2001) 71:841–50. 10.1097/00007890-200104150-00004 11349714

[34] Malacarne F Webster GJ Reignat S Gotto J Behboudi S Burroughs AK. Tracking the source of the hepatitis B virus-specific CD8 T cells during lamivudine treatment. J Infect Dis. (2003) 187:679–82. 10.1086/368369 12599086

[35] Demirkiran A Bosma BM Kok A Baan CC Metselaar HJ Ijzermans JN. Allosuppressive donor CD4+CD25+ regulatory T cells detach from the graft and circulate in recipients after liver transplantation. J Immunol. (2007) 178:6066–72. 10.4049/jimmunol.178.10.6066 17475831

[36] Moroso V Metselaar HJ Mancham S Tilanus HW Eissens D van der Meer A. Liver grafts contain a unique subset of natural killer cells that are transferred into the recipient after liver transplantation. Liver Transpl. (2010) 16:895–908. 10.1002/lt.22080 20583081

[37] Moroso V Famili F Papazian N Cupedo T van der Laan LJ Kazemier G. NK cells can generate from precursors in the adult human liver. Eur J Immunol. (2011) 41:3340–50. 10.1002/eji.201141760 21830211

[38] Segura E Valladeau-Guilemond J Donnadieu MH Sastre-Garau X Soumelis V Amigorena S. Characterization of resident and migratory dendritic cells in human lymph nodes. J Exp Med. (2012) 209:653–60. 10.1084/jem.20111457 22430490 PMC3328358

[39] Nizzoli G Krietsch J Weick A Steinfelder S Facciotti F Gruarin P. Human CD1c+ dendritic cells secrete high levels of IL-12 and potently prime cytotoxic T-cell responses. Blood. (2013) 122:932–42. 10.1182/blood-2013-04-495424 23794066

[40] Akila P Prashant V Suma MN Prashant SN Chaitra TR. CD163 and its expanding functional repertoire. Clin Chim Acta. (2012) 413:669–74. 10.1016/j.cca.2012.01.028 22309681

[41] Tu Z Bozorgzadeh A Pierce RH Kurtis J Crispe IN Orloff MS. TLR-dependent cross talk between human Kupffer cells and NK cells. J Exp Med. (2008) 205:233–44. 10.1084/jem.20072195 18195076 PMC2234385

[42] Krug A Towarowski A Britsch S Rothenfusser S Hornung V Bals R. Toll-like receptor expression reveals CpG DNA as a unique microbial stimulus for plasmacytoid dendritic cells which synergizes with CD40 ligand to induce high amounts of IL-12. Eur J Immunol. (2001) 31:3026–37. 10.1002/1521-4141(2001010)31:10<3026::AID-IMMU3026>3.0.CO;2-H 11592079

[43] Angel CE Chen CJ Horlacher OC Winkler S John T Browning J. Distinctive localization of antigen-presenting cells in human lymph nodes. Blood. (2009) 113:1257–67. 10.1182/blood-2008-06-165266 18987360 PMC2687552

[44] Marmey B Boix C Barbaroux JB Dieu-Nosjean MC Diebold J Audouin J. CD14 and CD169 expression in human lymph nodes and spleen: specific expansion of CD14+CD169- monocyte-derived cells in diffuse large B-cell lymphomas. Hum Pathol. (2006) 37:68–77. 10.1016/j.humpath.2005.09.016 16360418

[45] Mittag D Proietto AI Loudovaris T Mannering SI Vremec D Shortman K. Human dendritic cell subsets from spleen and blood are similar in phenotype and function but modified by donor health status. J Immunol. (2011) 186:6207–17. 10.4049/jimmunol.1002632 21515786

[46] Matsuno K Kudo S Ezaki T Miyakawa K. Isolation of dendritic cells in the rat liver lymph. Transplantation. (1995) 60:765–8. 10.1097/00007890-199510150-00027 7570992

[47] Sluijter BJ van den Hout MF Koster BD van Leeuwen PA Schneiders FL van de Ven R. Arming the melanoma sentinel lymph node through local administration of CpG-B and GM-CSF: recruitment and activation of BDCA3/CD141(+) dendritic cells and enhanced cross-presentation. Cancer Immunol Res. 3:495–505. 10.1158/2326-6066.CIR-14-0165 25633713

[48] Tanis W Mancham S Binda R Janssen HL Bezemer G JN IJ. Human hepatic lymph nodes contain normal numbers of mature myeloid dendritic cells but few plasmacytoid dendritic cells. Clin Immunol. (2004) 110:81–8. 10.1016/j.clim.2003.10.003 14962799

[49] Cella M Jarrossay D Facchetti F Alebardi O Nakajima H Lanzavecchia A. Plasmacytoid monocytes migrate to inflamed lymph nodes and produce large amounts of type I interferon. Nat Med. (1999) 5:919–23. 10.1038/11360 10426316

[50] Yoneyama H Matsuno K Zhang Y Nishiwaki T Kitabatake M Ueha S. Evidence for recruitment of plasmacytoid dendritic cell precursors to inflamed lymph nodes through high endothelial venules. Int Immunol. (2004) 16:915–28. 10.1093/intimm/dxh093 15159375

[51] Zhang S Kodys K Babcock GJ Szabo G. CD81/CD9 tetraspanins aid plasmacytoid dendritic cells in recognition of hepatitis C virus-infected cells and induction of interferon-alpha. Hepatology. (2013) 58:940–9. 10.1002/hep.25827 22577054 PMC4511847

[52] de Ruiter PE Boor PP de Jonge J Metselaar HJ Tilanus HW Ijzermans JN Prednisolone does not affect direct-acting antivirals against hepatitis C, but inhibits interferon-alpha production by plasmacytoid dendritic cells. Transpl Infect Dis. (2015) 17:707–15. 10.1111/tid.12430 26250892

[53] Yoneyama H Matsuno K Toda E Nishiwaki T Matsuo N Nakano A. Plasmacytoid DCs help lymph node DCs to induce anti-HSV CTLs. J Exp Med. (2005) 202:425–35. 10.1084/jem.20041961 16061729 PMC2213078

[54] Lou Y Liu C Kim GJ Liu YJ Hwu P Wang G. Plasmacytoid dendritic cells synergize with myeloid dendritic cells in the induction of antigen-specific antitumor immune responses. J Immunol. (2007) 178:1534–41. 10.4049/jimmunol.178.3.1534 17237402

[55] Savina A Amigorena S. Phagocytosis and antigen presentation in dendritic cells. Immunol Rev. (2007) 219:143–56. 10.1111/j.1600-065X.2007.00552.x 17850487

[56] Junt T Moseman EA Iannacone M Massberg S Lang PA Boes M. Subcapsular sinus macrophages in lymph nodes clear lymph-borne viruses and present them to antiviral B cells. Nature. (2007) 450:110–4. 10.1038/nature06287 17934446

[57] Hickman HD Takeda K Skon CN Murray FR Hensley SE Loomis J. Direct priming of antiviral CD8+ T cells in the peripheral interfollicular region of lymph nodes. Nat Immunol. (2008) 9:155–65. 10.1038/ni1557 18193049

[58] Asano K Nabeyama A Miyake Y Qiu CH Kurita A Tomura M. CD169-positive macrophages dominate antitumor immunity by crosspresenting dead cell-associated antigens. Immunity. (2011) 34:85–95. 10.1016/j.immuni.2010.12.011 21194983

[59] Gordon S Pluddemann A Mukhopadhyay S. Sinusoidal immunity: macrophages at the lymphohematopoietic interface. Cold Spring Harb Perspect Biol. (2014) 7:a016378. 10.1101/cshperspect.a016378 25502514 PMC4382741

[60] Palframan RT Jung S Cheng G Weninger W Luo Y Dorf M. Inflammatory chemokine transport and presentation in HEV: a remote control mechanism for monocyte recruitment to lymph nodes in inflamed tissues. J Exp Med. (2001) 194:1361–73. 10.1084/jem.194.9.1361 11696600 PMC2195988

